# APSified OCT-angiography analysis: Macula vessel density in healthy eyes during office hours

**DOI:** 10.1371/journal.pone.0282827

**Published:** 2023-03-09

**Authors:** Meike Müller, Julia Schottenhamml, Sami Hosari, Bettina Hohberger, Christian Y. Mardin

**Affiliations:** 1 Department of Ophthalmology, Friedrich-Alexander-Universität of Erlangen-Nürnberg, Erlangen, Bavaria, Germany; 2 Department of Computer Science, Friedrich-Alexander-Universität of Erlangen-Nürnberg, Erlangen, Bavaria, Germany; Tsukazaki Hospital, JAPAN

## Abstract

**Purpose:**

Optical coherence tomography angiography (OCT-A) can visualize retinal capillary microcirculation non-invasively. In order to investigate potential factors influencing OCT-A diagnostics, the aim of the present study was to determine circadian changes in macular vessel density (VD) in healthy adults during office hours, considering axial length (AL) and subfoveal choroidal thickness (CT).

**Methods:**

In the prospective study 30 eyes of 30 healthy subjects (mean age 28.7 ± 11.8, range 19–60 years) were recruited who underwent repeated measurements of AL, subfoveal CT and three-layer macula VD (superficial vascular plexus (SVP), intermediate capillary plexus (ICP) and deep capillary plexus (DCP)) on a single day at three predetermined timepoints (9 AM, 3 PM, and 9 PM). For better intra- and interindividual scan comparability, the new Anatomic Positioning System function (APS, part of Glaucoma Module Premium Edition [GMPE], Heidelberg Engineering, Germany) allowing analysis of identical retinal areas, was used for quantitative OCT-A analysis.

**Results:**

Overall mean macula VD was unchanged during office hours in SVP, ICP and DCP, respectively (p>0.05). In addition, AL and CT showed no statistically significant changes over time (p>0.05). Rather, a large interindividual variance of VD with different peak time was observed. Contrary to the overall data, sectorial VD changed in dependency of office hours in all layers with an increase of VD in SVP between 9 AM and 9 PM (p = 0.003), in ICP between 3 PM and 9 PM (p = 0.000), in DCP between 9 AM and 9 PM (p = 0.048), and 3 PM and 9 PM (p = 0.000), respectively.

**Conclusion:**

Overall mean macula VD, subfoveal CT and AL tended not to show statistically significant changes over time in this cohort, whereas a regional analysis of VD did. Therefore, a circadian influence on capillary microcirculation should be kept in mind. Moreover, the results highlight the importance of a more detailed analysis of VD in different sectors and different vascular layers. In addition, the pattern of diurnal variation could vary inter-individually, thus a patient-specific fluctuation pattern would need to be considered when evaluating these parameters in clinical practice.

## Introduction

Optical coherence tomography angiography (OCT-A) is a new non-invasive imaging technique, offering a novel diagnostic option by generating high-quality, three-dimensional images of the macular and peripapillary regions at capillary level [1,2]. In contrast to fundus fluorescein angiography (FA), the former gold standard to visualize retinochoroidal blood flow, OCT-A is based on detecting the reflection behavior of moving erythrocytes in a static environment and therefore no longer requires dye injection for vascular imaging as used in FA. In addition, FA is not able to distinguish between single retinal vascular layers [1,3].

OCT-A technology, which has been shown to have high correlation with anatomical structures [4], enables multi-layer analysis of vessel density (VD). Recent developments have made it possible to visualize three retinal sublayers with OCT-A: a superficial vascular plexus (SVP), intermediate capillary plexus (ICP) and deep capillary plexus (DCP) [5]. Since the macula with its complex microvasculature plays an important role in the pathogenesis of many ocular diseases and a reduction of its perfusion can be considered as an early glaucoma marker [6], a more detailed understanding of its regulation is the focus of clinical interest. In particular, when comparing longitudinal OCT-A data to study disease progression or to evaluate a therapeutic success, it is important to distinguish physiologic from pathologic factors that may influence alterations of VD, such as time of day. Circadian variations have already been described for parameters like axial length (AL), choroidal thickness (CT) or intraocular pressure (IOP) [7–11]. However, very little is known to date about diurnal fluctuations in retinal perfusion visualized by OCT-A, and so far there are only few published data [12–18].

To the best of our knowledge, the present study is the first to investigate potential circadian overall and sectorial macula VD changes of not only two but three retinal layers (including ICP) in healthy adult eyes during a time span from 9 AM to 9 PM. Additionally, axial length (AL) and subfoveal choroidal thickness (CT) changes were investigated.

## Material and methods

### Participants and study design

The present study was designed as a prospective control study. 30 eyes of 30 healthy adults were evaluated (7 male, 23 female). Mean age was 28.7 ± 11.8 years with a range of 19–60 years (women: 28.8 ± 11.4; men: 28.4 ± 13.0). According to the spherical equivalent refraction (SER), 15 emmetropic subjects (SER: +0.75 to -0.75 DS, mean: -0.13 ± 0.2 DS), 13 myopic (SER: ≤ -1.00 DS, mean: -3.46 ± 1.8 DS) and two hyperopic (SER: ≥ + 1.00 DS, mean +1.375 ± 0 DS) eyes were analyzed. A complete standardized ophthalmologic screening, including slit-lamp biomicroscopy, fundoscopy and Goldman applanation tonometry, was performed on each subject. The presence of any eye disease and previous ophthalmic surgery or laser treatment were exclusion criteria and IOP had to be within the normal range. The study protocol was approved by the local ethic committee of Erlangen and was performed in accordance with the tenets of the Declaration of Helsinki. Informed written consent, approved by the ethic committee of Erlangen, was obtained from all study participants.

### Image acquisition

All measurements were performed on each subject without prior pupil dilation at three consecutive sessions (9 AM, 3 PM and 9 PM) within one day. An interval of six hours had to be between each measurement. One eye of each subject was chosen randomly before the first acquisition. To avoid fluctuations in blood pressure and heart rate, the measurements were taken each time after a waiting period of ten minutes in a sitting position and the participants were prohibited to consume caffeine prior to each visit. Each session included a high-resolution three-layer en face OCT-A scan of macula region (including SVP, ICP and DCP) and a high-speed OCT scan with enhanced depth imaging mode (EDI) to determine subfoveal CT, both by Heidelberg Spectralis II OCT (Heidelberg Engineering, Heidelberg, Germany). In addition, AL was measured with IOL master 500 (Carl Zeiss Meditec AG, Jena, Germany). All OCT-A scans were recorded on a 2.9 x 2.9 mm^2^ window with a 15° x 15° angle and a lateral resolution of 5.7 μm/pixel. Subfoveal CT was measured manually with a vertical distance between the hyperreflective line of Bruch’s membrane and the choroid-scleral interface.

### Erlangen Angio-Tool and Anatomic Positioning System

After all OCT-A scans were manually checked for artefacts, shadows and correct segmentation, macula data was exported from the clinical database and then imported into the prototype SP-X1902 software (Heidelberg Engineering, Heidelberg, Germany). The Anatomic Positioning System function (APS, part of Glaucoma Module Premium Edition [GMPE], Heidelberg Engineering, Germany) allows each scan to be aligned to the patient´s individual Fovea to Bruch’s Membrane Opening (FoBMOC) axis for better intra- and interindividual scan comparability. Integration of APS information was also implemented into the Erlangen Angio-Tool (EA-Tool) version 2.0, coded in Matlab (The MathWorks, Inc., R2017b). In addition to the APS information, the macular en face OCT-A images of SVP, ICP and DCP were imported into the EA-Tool and analyzed separately for each scan. Overall and sectorial macula VD (12 sectors s1-s12 á 30°) were analyzed for SVP, ICP and DCP, respectively. The analyzed region of the scan size was 6.10 mm^2^.

### Statistical analysis

SPSS version 28 (IBM Corp. Released 2021. IBM SPSS Statistics for Windows, Version 28.0. Armonk, NY: IBM Corp.) was used for the statistical analysis and p-values less than 0.05 were considered to be statistically significant. Demographic information (age and gender) was available and used as covariates to correct for them. Moreover, interaction terms were firstly incorporated into the statistical model. After a first run, statistically not significant variables and interactions were removed and the model was run again without them. For all experiments, a linear mixed model with type III sum of squares was used with the daytime as predictor variable to examine circadian changes in VD as well as AL and CT. A random intercept was included into the model to account for a clustering in the patients. The daytime was set as a repeated measure with a compound symmetry covariance structure. For the sectorial VD analysis, the sectors were additionally added as a repeated measure with the same covariance structure. Pairwise comparisons were computed based on the estimated marginal means and were adjusted with Bonferroni to account for multiple comparisons. Macula VD, AL and CT data were presented as mean and standard deviation (SD).

## Results

### Macula OCT-A vessel density

Overall mean VD ± SD over time was 30.77 ± 1.6 (SVP), 22.97 ± 1.5 (ICP) and 24.44 ± 2.2 (DCP), respectively. A linear mixed model analysis with pairwise comparisons (9 AM to 3 PM, 9 AM to 9 PM and 3 PM to 9 PM*)* revealed no statistically significant changes in overall mean VD between the time points for all retinal layers (p>0.05). Table 1 presents overall mean VD of SVP, ICP, and DCP at the corresponding time of measurement and their respective p-values.

**Table 1:** Overall mean vessel density with standard deviation for each retinal sublayer at the corresponding time of measurement and respective p-values showing no statistically significant diurnal changes.

	9 AM mean VD ± SD	3 PM mean VD ± SD	9 PM mean VD ± SD	p		
				9 AM– 3 PM	9 AM– 9 PM	3 PM– 9 PM
**SVP**	30.59 ± 1.7	30.75 ± 1.7	30.97 ± 1.4	1.00	0.19	0.76
**ICP**	22.96 ± 1.6	22.72 ± 1.7	23.22 ± 1.2	0.88	0.80	0.10
**DCP**	24.39 ± 2.2	24.17 ± 2.2	24.76 ± 2.1	1.00	0.57	0.13

VD, vessel density; standard deviation, SD; SVP, superficial vascular plexus; ICP, intermediate capillary plexus; DCP, deep capillary plexus. P-values were generated from pairwise comparisons (9 AM to 3 PM, 9 AM to 9 PM and 3 PM to 9 PM). P-values of p<0.05 were considered statistically significant.

**Fig 1:**
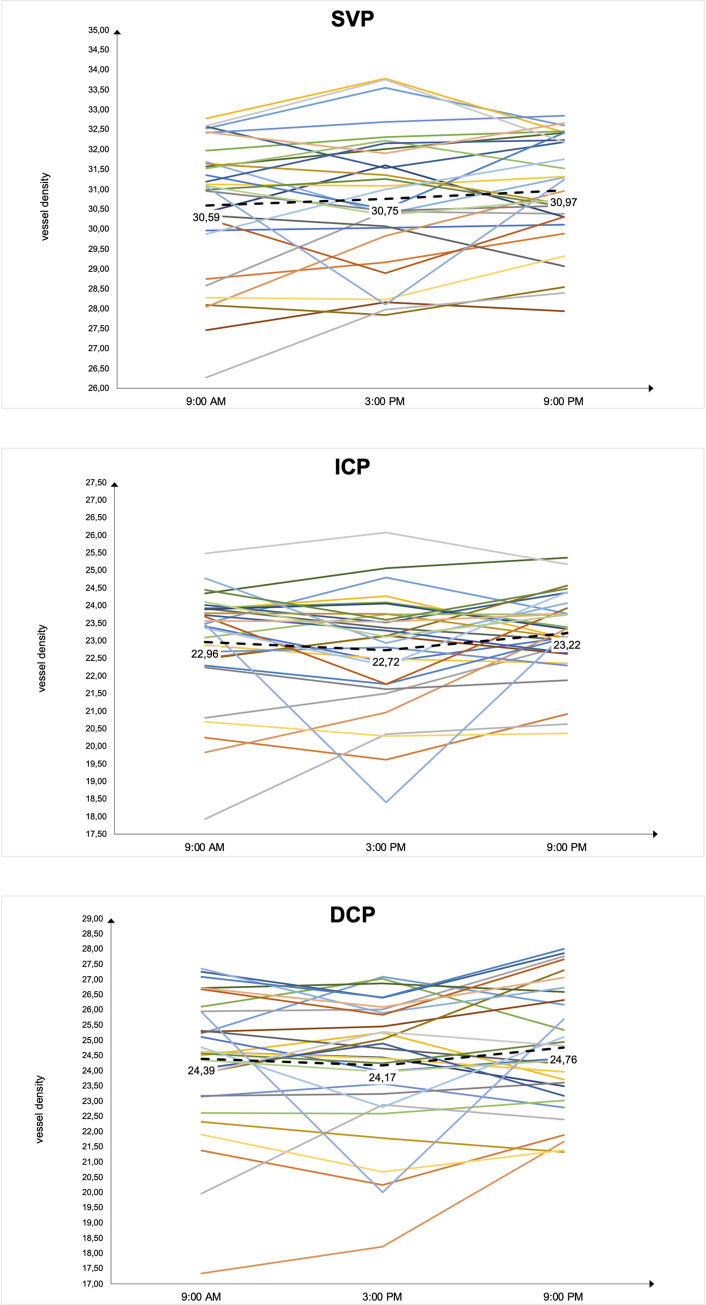
Circadian vessel density curves showing patient-specific fluctuations with different peak times. Overall vessel density (VD) curves are presented for superficial vascular plexus (SVP), intermediate capillary plexus (ICP) and deep capillary plexus (DCP) at 9 AM, 3 PM and 9 PM. The *dashed line* corresponds to the respective mean values.

While overall mean VD remained constant, it was found that the individual circadian VD curves differed from subject to subject. No uniform fluctuation pattern but a high interindividual variance with different peak times was determined (Fig 1).

Contrary to overall data, sectorial VD analysis revealed statistically significant changes between the selected time points in all sectors. With pairwise comparisons of linear mixed model analyses, statistically significant changes with an increase of VD in all sectors in SVP were observed between the 9 AM and 9 PM measurement (p = 0.003). For ICP, VD increased statistically significant in all sectors from the 3 PM to 9 PM measurement (p = 0.000). Analysis of VD data of DCP, a statistically significant increase between the 9 AM and 9 PM measurement (p = 0.048) and 3 PM and 9 PM (p = 0.000) was observed, respectively. All p-values from pairwise comparisons are shown in Table 2.

**Table 2:** P-values generated from pairwise comparisons of mixed model analysis showing significant changes of mean sectorial VD in all 12 sectors between the measurement times for each retinal sublayer.

	9 AM– 9 PM	9 AM– 3 PM	3 PM– 9 PM
SVP	***0*.*003***	0.517	0.177
ICP	0.084	0.112	***0*.*000***
DCP	***0*.*048***	0.456	***0*.*000***

SVP, superficial vascular plexus; ICP, intermediate capillary plexus; DCP, deep capillary plexus. P-values were generated from pairwise comparisons (9 AM to 3 PM, 9 AM to 9 PM and 3 PM to 9 PM) and relate to all 12 sectors. P-values of p<0.05 were considered statistically significant and are ***highlighted***.

In addition, VD varied between all sectors: pairwise comparisons of linear mixed model analyses revealed multiple statistically significant differences of VD between the sectors for each layer and for each measurement time (p<0.05). Mean sectorial VD ± SD (sector 1–12) of each layer at the corresponding time of measurement are shown in Table 3.

**Table 3:** Sectorial mean vessel density with standard deviation for each retinal sublayer at the corresponding time of measurement.

	SVP			ICP			DCP		
	mean VD ± SD			mean VD ± SD			mean VD ± SD		
	9 AM	3 PM	9 PM	9 AM	3 PM	9 PM	9 AM	3 PM	9 PM
**s1**	31.19 ±2.6	31.50 ±2.1	31.52 ±2.1	22.64 ±2.4	22.25 ±2.7	22.58 ±2.3	24.71 ±3.3	24.36 ±3.8	25.11 ±3.3
**s2**	30.34 ±2.4	30.57 ±2.5	30.80 ±2.3	22.84 ±2.4	22.17 ±2.7	22.55 ±2.5	24.61 ±3.0	23.94 ±3.7	24.64 ±3.3
**s3**	30.00 ±2.3	30.01 ±3.4	30.32 ±2.4	24.07 ±2.3	23.43 ±3.2	24.16 ±1.8	24.93 ±3.4	24.25 ±4.1	25.20 ±2.6
**s4**	30.62 ±2.2	30.53 ±2.5	30.70 ±2.3	23.87 ±2.2	23.58 ±2.6	24.10 ±1.7	25.27 ±3.0	24.98 ±3.2	25.47 ±2.4
**s5**	30.80 ±1.9	31.21 ±2.2	31.58 ±1.8	22.40 ±2.2	22.54 ±2.5	23.08 ±1.9	23.85 ±2.6	24.23 ±2.9	24.59 ±3.0
**s6**	31.31 ±3.1	31.73 ±2.0	32.05 ±1.7	21.94 ±2.6	22.52 ±2.0	22.95 ±1.9	23.95 ±3.2	24.67 ±2.9	24.75 ±2.9
**s7**	31.93 ±2.3	32.06 ±1.7	32.17 ±1.7	22.21 ±2.4	22.31 ±2.4	22.90 ±1.8	23.91 ±3.0	24.02 ±3.0	24.67 ±2.8
**s8**	31.06 ±1.9	31.16 ±1.9	31.34 ±1.7	22.42 ±2.1	22.64 ±2.1	23.10 ±1.6	23.23 ±2.9	23.86 ±2.4	24.37 ±2.6
**s9**	29.82 ±1.6	29.62 ±2.4	29.82 ±1.7	23.76 ±1.4	23.30 ±1.9	23.94 ±1.9	24.55 ±2.9	24.23 ±2.6	24.29 ±3.0
**s10**	28.37 ±2.2	28.57 ±2.5	28.83 ±1.7	23.88 ±1.7	22.99 ±2.5	23.78 ±1.4	24.33 ±2.3	23.41 ±2.9	24.14 ±2.7
**s11**	30.19 ±2.2	30.39 ±1.9	30.71 ±1.7	23.02 ±1.8	22.56 ±1.7	22.93 ±1.6	24.57 ±2.4	23.83 ±2.7	24.84 ±2.6
**s12**	31.47 ±2.7	31.69 ±2.4	31.82 ±2.1	22.52 ±2.5	22.40 ±2.4	22.53 ±2.0	24.79 ±3.0	24.31 ±3.5	25.00 ±2.9

VD, vessel density; standard deviation, SD; SVP, superficial vascular plexus; ICP, intermediate capillary plexus; DCP, deep capillary plexus; s1-s12, sector 1–12.

### Axial length and subfoveal choroidal thickness

Mean AL *±* SD was 23.96 *±* 0.79 mm (range 23.67–24.26 mm) and mean subfoveal CT *±* SD was 332.99 *±* 78.1 μm (range 303.15–362.99 μm) over time. Linear mixed model analyses with pairwise comparisons (9 AM to 3 PM, 9 AM to 9 PM and 3 PM to 9 PM) were done to reveal diurnal changes. Both AL and CT showed no statistically significant changes between the measurement times (AL: p = 1.00; CT: p = 1.00), yet a wide SD of the values was observed. Table 4 provides an overview of mean AL *±* SD and subfoveal CT *±* SD at the corresponding time of measurement together with the respective p-values.

**Table 4:** Mean axial length and subfoveal choroidal thickness with standard deviation at the corresponding time of measurement and respective p-values showing no statistically significant diurnal changes.

	9 AM mean ± SD	3 PM mean ± SD	9 PM mean ± SD	p
**AL**	23.963 ± 0.80	23.964 ± 0.80	23.963 ± 0.80	1.00
**CT**	332.83 ± 77.9	332.0 ± 79.6	334.13 ± 79.2	1.00

AL, axial length in mm; CT, subfoveal choroidal thickness in μm; SD, standard deviation. P-values were generated from pairwise comparisons (9 AM to 3 PM, 9 AM to 9 PM and 3 PM to 9 PM). P-values of p<0.05 were considered statistically significant.

## Discussion

OCT-A technology enables three-dimensional multi-layer imaging of retinochoroidal vascular structures in detail [1,2]. With the capability to generate high-resolution scans efficiently and non-invasively, OCT-A is becoming increasingly important in the diagnosis of ocular vascular diseases [19]. For proper use, physiological factors or drugs affecting retinochoroidal blood flow are clinically meaningful in order to be reliably distinguished from real pathological changes, especially when evaluating longitudinal OCT-A data. Yet, the effect of physical exertion, age, sex, smoking and mydriatic agents on retinal perfusion have been described [20–23]. This study aimed to investigate circadian variations of retinal macula VD measured with OCT-A in healthy adults for a better understanding of the complex functioning and control of capillary structures in the retina. Additionally, AL and CT changes were investigated.

Overall mean VD did not change significantly between 9 AM to 9 PM for SVP, ICP and DCP in the present cohort. AL and CT showed no statistically significant diurnal variations either. A uniform fluctuation pattern of overall mean VD of all subjects was not detectable, but rather a hint of patient-specific fluctuation with high interindividual variance and different peak times. Contrary to overall VD, a sectorial analysis revealed statistically significant changes in all layers, showing a trend of VD to increase over the day. For SVP, a statistically significant increase of VD was seen between the 9 AM measurement and the 9 PM measurement (p = 0.003), for ICP between 3 PM and 9 PM (p = 0.000) and for DCP between 9 AM and 9 PM (p = 0.048) as well as 3 PM and 9 PM (p = 0.000), respectively.

The first step for correct scan comparability resulting in high diagnostic value, is precise data generation and analysis. Since there are different OCT-A devices and no standardized analysis software is available so far, different perfusion values can be generated. Moreover, high scan quality is required as it affects longitudinal changes of VD. Increasing scan quality has been shown to correlate with increasing peripapillary and macula VD [12,24]. In the present study all scans were performed with en face OCT-A module by Heidelberg Spectralis II OCT. It provides detailed visualization of fine capillary vascular networks within three retinochoroidal layers (SVP, ICP and DCP) with high lateral and axial resolution. By using TruTrack Active Eye Tracking® and a projection artefact removal (PAR) algorithm, which uses information from the superficial vascular complex to remove artefacts and shadows out of underlying layers, microvascular structures can be imaged with high precision. In addition with the semi-automated EA-Tool, which enables quantification of VD with a high level of reliability and reproducibility [25], even smallest capillary changes can be displayed.

**Fig 2:**
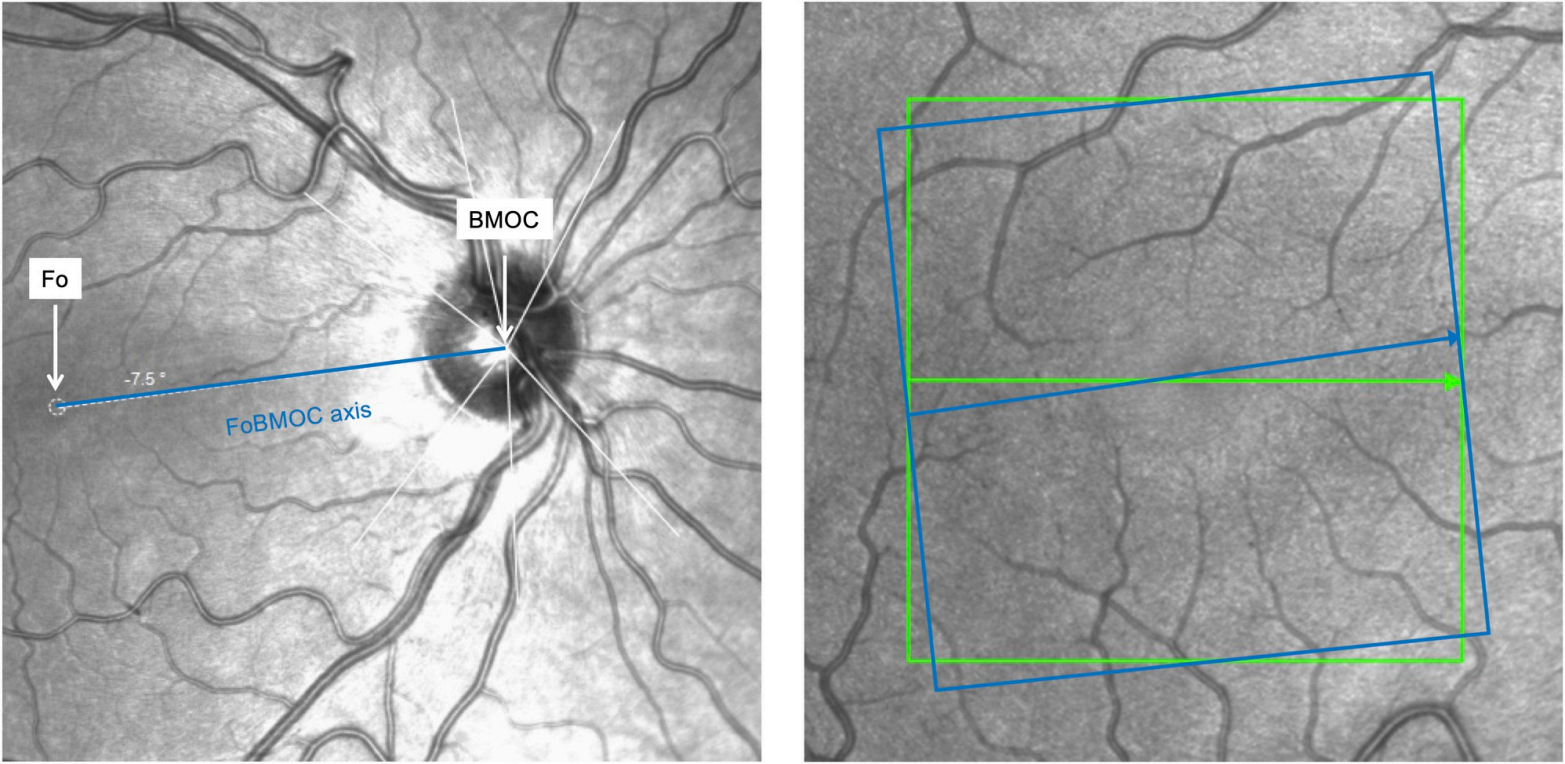
OCT-A scan window of macula region without (green box) and after (blue box) aligning according to the fovea (Fo) to Bruch’s membrane opening center (BMOC) axis (FoBMOC axis).

Furthermore, all measurements require a reliable point-to-point comparison to avoid variations in VD due to site-specific variations. So far, all scans of a single patient can be aligned to the baseline scan using the standard follow-up mode to eliminate influence of head tilt and eye rotation. For a more accurate scan comparison not only within a subject but also between different subjects, it must be considered that interindividual anatomic variations exist and may affect sectorial analysis. The new APS tool enables improved analysis of identical retinal areas by defining two anatomical landmarks, the fovea (Fo) and the Bruch’s membrane opening center (BMOC) on an optical nerve head (ONH) circle scan. Afterwards, the OCT-A scan can be re-positioned according to the FoBMOC axis by aligning the B-scan axis parallel to the FoBMOC axis (Fig 2).

Up to now little is known about diurnal changes of retinal perfusion. In this publication, no statistically significant overall mean VD changes were found during predetermined office hours between 9 AM to 9 PM. Consistent with these findings, previous studies have described a constant retinal blood flow in healthy subjects throughout the day [13–16]. Odabaş et al. found no statistically significant changes in VD for superficial and deep retinal layer (except for superior zone of the deep retinal layer) between 9 AM and 3 PM [13]. Lin et al., who repeated their scan sessions between 9 AM and 5 PM on a separate day to achieve highly reliable results, could not detect significant circadian variations either [15]. Similar results of a constant sublayer and full retinal perfusion measured at 7 AM and 4 PM on a single day were achieved by Rommel et al. Interestingly, the authors found out that retinal blood flow was unaffected by mean arterial pressure and IOP which has led them to assume that the insensitivity may be caused by autoregulatory mechanisms [16]. While this effect may account for the retina, the choriocapillaris has found to behave differently. Significant diurnal changes of choroidal microperfusion were found in healthy individuals [26–28] and also in patients with idiopathic epiretinal membrane [29]. Moreover, previous studies have shown that choroidal blood flow, unlike retinal blood flow, depends on changes in systemic pressure [28,30,31]. Yet, the exact mechanisms are not totally understood and further investigations are necessary. While vascular structures in the choroid were found to be rich of autonomic vasoactive nerve endings, adrenergic fibers in the retina were detected between inner nuclear and inner plexiform layers only in areas until the lamina cribrosa [32,33]. Blood flow in peripheral retinal areas is thought to be subject to autoregulation, which is mediated by an interaction of myogenic and metabolic factors through a release of vasoactive substances [33–35]. Changes in perfusion pressure or metabolic demand of the tissue cause an adjustment of the vascular tone of the resistance vessels by influencing arteriolar smooth muscle cells and capillary pericytes [35].

While this mechanism works well in healthy eyes, there is evidence that autoregulatory capacity is impaired in patients with ocular diseases such as diabetic retinopathy [36] and glaucoma [12,14,17]. Mansouri et al. reported diurnal variations of peripapillary and macula VD with a small but mostly not significant increase throughout their scan sessions [12]. Baek et al. showed significantly greater changes of peripapillary and macula VD as well as IOP and mean ocular-perfusion pressure in eyes with primary open-angle glaucoma than in healthy ones [14]. Müller et al. found a statistically significant increase in macula VD for deep layer and a relationship between VD and mean arterial pressure as well as heart rate [17]. Thus, based on the assumption of disturbed blood flow regulation in those patients, repeated OCT-A measurements need to be performed every time under the same conditions and at the same time of day.

In contrast to overall mean VD, this study showed statistically significant differences in all layers in regional analysis of VD by subdivision into 12 sectors in the present cohort. A possible reason for this could be the large spread of the individual (as seen in Fig 1) as well as the sectorial (as seen in Table 3) values around the mean. Accordingly, a calculation of overall mean VD would not represent the actual conditions. Therefore, a subdivision into sectors as well as vascular layers might be considered for analysis of VD.

Previous studies have reported that AL and CT exhibit circadian fluctuations in healthy subjects [8,9,11,37–39]. Using partial coherence interferometry (PCI), Stone et al. proved a significant diurnal variation of AL with a maximum ad midday [39]. Brown et al., who also used PCI, were the first to show diurnal variations of CT and described a trend of AL and CT to fluctuate in antiphase [37]. Chakraborty et al. also showed nearly antiphasic variations in mean AL and mean CT [8]. Controversially to the findings of Tan et al., Usui et al. and Lee et al., who reported a significant decrease in CT during the day (maximum in the morning and minimum in the evening) [9,11,38], Chakraborty et al. found the choroid to be thinnest in the morning and thicker at night [8]. A possible reason could be that their measurements were performed with an optical biometer which may not be comparable to OCT. Furthermore, it was determined that both myopes and participants with longer AL and thinner CT had a significantly lower pattern of diurnal variation with lower amplitude [9,38]. In contrast, the present study did not detect significant diurnal changes between 9 AM and 9 PM. But since almost half of all participants in this study were myopic (13 of 30), previous findings of a lower fluctuation pattern in myopes may explain why no variations were detected. Furthermore, no uniform fluctuation pattern but a wide SD of the values was observed. This finding is consistent with study data from Pollithy et al., who also found no significant circadian CT changes with Heidelberg Spectralis OCT, but individually different fluctuation patterns within a 24-hour period [40]. Another study by Osmanbasoglu et al., which also used the Heidelberg Spectralis OCT, could not detect significant mean CT changes in healthy eyes between 9 AM and 4 PM either [41]. A possible reason for the differing results could be a mix of factors, including differences between devices or time span of the measurements, which did not include night times, while others covered 24 hours or even longer. As previously described by Pollithy et al. [40], no uniform increase or decrease in the values of all subjects was observed in the present study. However, the statements of most of the authors are based on calculated mean values at the respective time points [8,9,11,38], so interindividual differences could not be taken into account.

This study is not without limitations. The selected timepoints did not contain night times, so the results do not show whole diurnal fluctuation. Moreover, only one OCT-A device was used, but as mentioned above, VD might differ between devices. Advantage of a small scan size area of 2.9x2.9 mm^2^ is generating high-resolution OCT-A images, but as a result, a large retinal area remains unclear. Furthermore, the cohort of the present study is small, thus the results of this paper should be seen as trend, which has to be validated in studies with larger patients’ cohorts. Therefore, further studies with larger patient database are necessary. In addition, systemic factors such as IOP, blood pressure or hydration were not recorded, so their influence on diurnal retinal blood flow could not be investigated. This may be interesting when assuming that retinal VD might be subject to any autoregulation. Finally, it remains to be seen whether peripapillary region shows circadian fluctuations as macula was at the center of interest in this study.

## Conclusion

As a non-invasive tool for imaging and evaluating capillary retinochoroidal blood flow, OCT-A expands diagnostic spectrum. This is the first study evaluating diurnal fluctuations of three-layer retinal macular VD in healthy adult eyes. Although overall macula VD, CT and AL showed no significant changes during office hours, regional differences and an interindividual pattern of diurnal fluctuation were observed and should be considered in clinical routine. However, since the exact mechanism and the influence of various factors on retinal microcirculation are not totally understood at this stage, the study results should be seen as first hints and need to be confirmed by further investigations in larger patients’ cohorts.

## Supporting information

S1 Dataset (DOCX)
