# Supplementary material for: APSified OCT-angiography analysis: Macula vessel density in healthy eyes during office hours
Source: PLoS One. 2023 Mar 9;18(3):e0282827. doi: 10.1371/journal.pone.0282827 (PMC9997993; doi:10.1371/journal.pone.0282827)
Supplement: S1 Dataset — (DOCX) [file pone.0282827.s001.docx]

S1 Dataset. Overview of the underlying raw data of the study of axial length, choroidal thickness and vessel density presented in separate tables (S1A-N).

S1A Table. Axial length

| **Patient** | **OD/ OS** | **Axial length [mm]** | | |
| --- | --- | --- | --- | --- |
|  |  | **9 AM** | **3 PM** | **9 PM** |
| 1 | 1 | 24,96 | 24,97 | 24,97 |
| 2 | 0 | 22,66 | 22,67 | 22,66 |
| 3 | 1 | 24,49 | 24,47 | 24,47 |
| 4 | 1 | 23 | 23,01 | 23 |
| 5 | 1 | 23,07 | 23,07 | 23,08 |
| 6 | 0 | 23,31 | 23,3 | 23,31 |
| 7 | 0 | 24,11 | 24,12 | 24,12 |
| 8 | 0 | 24,6 | 24,6 | 24,59 |
| 9 | 1 | 22,8 | 22,81 | 22,8 |
| 10 | 0 | 23,34 | 23,35 | 23,34 |
| 11 | 1 | 25,64 | 25,64 | 25,64 |
| 12 | 0 | 23,69 | 23,68 | 23,69 |
| 13 | 0 | 24,29 | 24,29 | 24,29 |
| 14 | 1 | 23,28 | 23,27 | 23,28 |
| 15 | 1 | 23,9 | 23,9 | 23,9 |
| 16 | 1 | 24,14 | 24,15 | 24,14 |
| 17 | 0 | 24,18 | 24,18 | 24,18 |
| 18 | 1 | 24,18 | 24,18 | 24,18 |
| 19 | 1 | 24,4 | 24,39 | 24,39 |
| 20 | 1 | 24,55 | 24,55 | 24,55 |
| 21 | 1 | 24,22 | 24,23 | 24,24 |
| 22 | 1 | 24,79 | 24,81 | 24,8 |
| 23 | 0 | 24,12 | 24,12 | 24,12 |
| 24 | 1 | 22,71 | 22,71 | 22,71 |
| 25 | 1 | 24,09 | 24,08 | 24,08 |
| 26 | 0 | 23,66 | 23,67 | 23,67 |
| 27 | 0 | 23,19 | 23,2 | 23,19 |
| 28 | 1 | 23,52 | 23,51 | 23,5 |
| 29 | 0 | 25,98 | 25,98 | 25,97 |
| 30 | 0 | 24,02 | 24,02 | 24,02 |

0= OD, right eye; 1= OS, left eye.

S1B Table. Choroidal thickness

| **Patient** | **OD/ OS** | **Choroidal thickness [μm]** | | |
| --- | --- | --- | --- | --- |
|  |  | **9 AM** | **3 PM** | **9 PM** |
| 1 | 1 | 362 | 357 | 370 |
| 2 | 0 | 458 | 452 | 456 |
| 3 | 1 | 277 | 273 | 275 |
| 4 | 1 | 358 | 375 | 358 |
| 5 | 1 | 395 | 399 | 382 |
| 6 | 0 | 432 | 440 | 438 |
| 7 | 0 | 417 | 410 | 409 |
| 8 | 0 | 314 | 310 | 320 |
| 9 | 1 | 398 | 384 | 407 |
| 10 | 0 | 312 | 302 | 320 |
| 11 | 1 | 181 | 181 | 183 |
| 12 | 0 | 286 | 286 | 288 |
| 13 | 0 | 464 | 463 | 450 |
| 14 | 1 | 362 | 364 | 365 |
| 15 | 1 | 356 | 356 | 356 |
| 16 | 1 | 222 | 221 | 215 |
| 17 | 0 | 238 | 239 | 238 |
| 18 | 1 | 375 | 382 | 386 |
| 19 | 1 | 315 | 326 | 328 |
| 20 | 1 | 362 | 396 | 399 |
| 21 | 1 | 255 | 238 | 246 |
| 22 | 1 | 253 | 235 | 237 |
| 23 | 0 | 249 | 247 | 246 |
| 24 | 1 | 443 | 434 | 439 |
| 25 | 1 | 202 | 203 | 202 |
| 26 | 0 | 398 | 390 | 393 |
| 27 | 0 | 331 | 329 | 331 |
| 28 | 1 | 349 | 342 | 359 |
| 29 | 0 | 248 | 245 | 247 |
| 30 | 0 | 373 | 381 | 381 |

0= OD, right eye; 1= OS, left eye.

S1C Table. Overall vessel density of all sectors for superficial vascular plexus

| **Patient** | **OD/ OS** | **Overall vessel density** | | |
| --- | --- | --- | --- | --- |
|  |  | **9 AM** | **3 PM** | **9 PM** |
| 1 | 1 | 32,53 | 33,54 | 32,60 |
| 2 | 0 | 28,74 | 29,17 | 29,89 |
| 3 | 1 | 28,58 | 30,43 | 30,38 |
| 4 | 1 | 32,77 | 33,78 | 32,42 |
| 5 | 1 | 29,96 | 30,03 | 30,11 |
| 6 | 0 | 31,96 | 32,31 | 32,46 |
| 7 | 0 | 32,57 | 31,53 | 32,18 |
| 8 | 0 | 27,46 | 28,17 | 27,93 |
| 9 | 1 | 30,35 | 30,07 | 29,07 |
| 10 | 0 | 28,10 | 27,84 | 28,54 |
| 11 | 1 | 30,42 | 31,60 | 30,30 |
| 12 | 0 | 31,57 | 32,01 | 32,42 |
| 13 | 0 | 31,68 | 30,38 | 31,29 |
| 14 | 1 | 28,04 | 29,82 | 30,96 |
| 15 | 1 | 26,28 | 27,97 | 28,39 |
| 16 | 1 | 31,12 | 31,08 | 31,31 |
| 17 | 0 | 32,42 | 32,69 | 32,84 |
| 18 | 1 | 31,52 | 32,22 | 31,52 |
| 19 | 1 | 31,35 | 30,49 | 32,41 |
| 20 | 1 | 30,26 | 28,89 | 30,30 |
| 21 | 1 | 30,96 | 30,46 | 30,58 |
| 22 | 1 | 31,64 | 31,35 | 30,62 |
| 23 | 0 | 31,19 | 32,15 | 32,23 |
| 24 | 1 | 30,99 | 31,26 | 30,57 |
| 25 | 1 | 29,87 | 31,00 | 31,75 |
| 26 | 0 | 32,44 | 31,90 | 32,66 |
| 27 | 0 | 32,59 | 33,75 | 32,18 |
| 28 | 1 | 28,27 | 28,23 | 29,32 |
| 29 | 0 | 31,06 | 28,10 | 31,24 |
| 30 | 0 | 31,08 | 30,35 | 30,74 |

0= OD, right eye; 1= OS, left eye.

S1D Table. Overall vessel density of all sectors for intermediate capillary plexus

| **Patient** | **OD/ OS** | **Overall vessel density** | | |
| --- | --- | --- | --- | --- |
|  |  | **9 AM** | **3 PM** | **9 PM** |
| 1 | 1 | 23,49 | 24,80 | 23,77 |
| 2 | 0 | 20,25 | 19,61 | 20,92 |
| 3 | 1 | 20,81 | 21,51 | 22,88 |
| 4 | 1 | 23,90 | 24,26 | 23,06 |
| 5 | 1 | 23,41 | 22,39 | 23,09 |
| 6 | 0 | 23,92 | 24,09 | 23,39 |
| 7 | 0 | 24,01 | 23,51 | 24,36 |
| 8 | 0 | 22,49 | 23,14 | 22,63 |
| 9 | 1 | 23,92 | 23,37 | 23,02 |
| 10 | 0 | 22,51 | 23,13 | 24,57 |
| 11 | 1 | 23,90 | 24,06 | 23,32 |
| 12 | 0 | 24,34 | 25,06 | 25,35 |
| 13 | 0 | 24,78 | 22,94 | 24,06 |
| 14 | 1 | 19,82 | 20,96 | 23,34 |
| 15 | 1 | 17,93 | 20,34 | 20,63 |
| 16 | 1 | 22,86 | 22,48 | 22,36 |
| 17 | 0 | 22,68 | 22,81 | 22,29 |
| 18 | 1 | 23,08 | 23,75 | 23,75 |
| 19 | 1 | 22,29 | 21,76 | 23,09 |
| 20 | 1 | 23,68 | 21,77 | 23,93 |
| 21 | 1 | 22,24 | 21,62 | 21,88 |
| 22 | 1 | 23,79 | 23,76 | 23,07 |
| 23 | 0 | 23,73 | 23,28 | 22,66 |
| 24 | 1 | 24,44 | 23,60 | 24,47 |
| 25 | 1 | 23,37 | 22,30 | 24,38 |
| 26 | 0 | 23,57 | 23,54 | 23,76 |
| 27 | 0 | 25,48 | 26,08 | 25,17 |
| 28 | 1 | 20,70 | 20,29 | 20,36 |
| 29 | 0 | 23,47 | 18,40 | 23,25 |
| 30 | 0 | 24,10 | 23,11 | 23,73 |

0= OD, right eye; 1= OS, left eye.

S1E Table. Overall vessel density of all sectors for deep capillary plexus

| **Patient** | **OD/ OS** | **Overall vessel density** | | |
| --- | --- | --- | --- | --- |
|  |  | **9 AM** | **3 PM** | **9 PM** |
| 1 | 1 | 25,23 | 27,08 | 26,16 |
| 2 | 0 | 21,38 | 20,24 | 21,88 |
| 3 | 1 | 25,94 | 26,01 | 27,76 |
| 4 | 1 | 24,50 | 25,25 | 23,70 |
| 5 | 1 | 25,11 | 23,98 | 24,41 |
| 6 | 0 | 26,10 | 27,02 | 25,34 |
| 7 | 0 | 27,24 | 26,40 | 27,86 |
| 8 | 0 | 25,27 | 25,46 | 26,32 |
| 9 | 1 | 25,31 | 24,73 | 24,22 |
| 10 | 0 | 23,92 | 25,04 | 27,30 |
| 11 | 1 | 24,61 | 24,43 | 23,48 |
| 12 | 0 | 26,71 | 26,87 | 26,58 |
| 13 | 0 | 27,35 | 25,89 | 26,73 |
| 14 | 1 | 17,34 | 18,21 | 21,67 |
| 15 | 1 | 19,96 | 22,87 | 22,40 |
| 16 | 1 | 24,61 | 24,41 | 23,96 |
| 17 | 0 | 23,14 | 23,56 | 22,79 |
| 18 | 1 | 22,61 | 22,58 | 23,02 |
| 19 | 1 | 27,08 | 26,40 | 28,00 |
| 20 | 1 | 26,67 | 25,83 | 27,65 |
| 21 | 1 | 23,17 | 23,23 | 23,60 |
| 22 | 1 | 22,32 | 21,79 | 21,32 |
| 23 | 0 | 24,06 | 24,89 | 23,17 |
| 24 | 1 | 24,55 | 24,24 | 24,94 |
| 25 | 1 | 24,76 | 22,80 | 25,10 |
| 26 | 0 | 26,71 | 26,09 | 27,06 |
| 27 | 0 | 23,91 | 25,28 | 24,82 |
| 28 | 1 | 21,90 | 20,68 | 21,39 |
| 29 | 0 | 25,92 | 20,00 | 25,69 |
| 30 | 0 | 24,35 | 23,96 | 24,34 |

0= OD, right eye; 1= OS, left eye.

S1F Table. 9 AM sectorial vessel density for superficial vascular plexus

| **Patient** | **OD/**  **OS** | **s1** | **s2** | **s3** | **s4** | **s5** | **s6** | **s7** | **s8** | **s9** | **s10** | **s11** | **s12** |
| --- | --- | --- | --- | --- | --- | --- | --- | --- | --- | --- | --- | --- | --- |
| 1 | 1 | 35,32 | 32,5 | 31,64 | 31,46 | 33,29 | 33,63 | 32,88 | 32,24 | 30,95 | 31,08 | 30,45 | 34,86 |
| 2 | 0 | 24,85 | 26,25 | 27,57 | 31,12 | 29,05 | 31,12 | 31,81 | 32,32 | 30,24 | 26,89 | 27,67 | 26,03 |
| 3 | 1 | 27,99 | 28 | 29,58 | 30,23 | 30,72 | 29,69 | 29,42 | 30,48 | 29,14 | 27,87 | 25,1 | 24,74 |
| 4 | 1 | 32,22 | 33,81 | 29,76 | 31,21 | 32,73 | 35,75 | 34,92 | 34,65 | 31,72 | 30,47 | 32,27 | 33,76 |
| 5 | 1 | 31,98 | 28,81 | 28,52 | 30,53 | 31,16 | 30,02 | 31,1 | 30,48 | 29,84 | 26,77 | 29,65 | 30,7 |
| 6 | 0 | 30,87 | 32,97 | 34,09 | 35,42 | 30,02 | 32,5 | 33,09 | 33,43 | 30,18 | 29,04 | 31,01 | 30,95 |
| 7 | 0 | 34,58 | 32,87 | 32,59 | 31,87 | 32,38 | 33,78 | 34,28 | 32,72 | 30,6 | 27,55 | 32,41 | 35,17 |
| 8 | 0 | 29,47 | 26,14 | 25,78 | 26,12 | 27,51 | 30,1 | 29,69 | 28,91 | 25,29 | 25,64 | 26,7 | 28,16 |
| 9 | 1 | 28,29 | 28,93 | 27,8 | 29,92 | 31,43 | 29,74 | 35,03 | 30,88 | 30,15 | 29,84 | 30,32 | 31,81 |
| 10 | 0 | 27,42 | 27,42 | 29,16 | 28,2 | 28,45 | 28,34 | 32,13 | 29,09 | 28,74 | 24,17 | 25,98 | 28,04 |
| 11 | 1 | 31,77 | 30,72 | 32,03 | 32,66 | 30,33 | 31,59 | 29,17 | 27,47 | 28,61 | 28,49 | 30,7 | 31,49 |
| 12 | 0 | 31,31 | 30,22 | 31,86 | 32,68 | 29,51 | 29,36 | 32,34 | 33,78 | 32,43 | 29,82 | 31,85 | 33,7 |
| 13 | 0 | 34,34 | 32,93 | 28,99 | 30,96 | 31,98 | 33,46 | 35 | 30,94 | 29,26 | 29,67 | 30,57 | 32,08 |
| 14 | 1 | 25,18 | 29,77 | 22,7 | 26,95 | 27,29 | 30,65 | 29,31 | 29,18 | 28,36 | 27,02 | 30,39 | 29,68 |
| 15 | 1 | 29,17 | 26,56 | 28,78 | 27,72 | 27,08 | 19,27 | 26,21 | 25,35 | 28,8 | 24,89 | 25,19 | 26,28 |
| 16 | 1 | 32,52 | 31,05 | 28,51 | 28,96 | 32,24 | 32,81 | 32,97 | 32,15 | 28,44 | 27,79 | 32,12 | 33,89 |
| 17 | 0 | 31,8 | 33,81 | 32,47 | 33,44 | 32,07 | 33,44 | 32,62 | 32,44 | 32,08 | 31,79 | 31,99 | 31,04 |
| 18 | 1 | 31,34 | 30,59 | 30,54 | 31,58 | 32,82 | 30,66 | 33,33 | 32,22 | 31,47 | 29,33 | 32,29 | 32,11 |
| 19 | 1 | 30,99 | 33,26 | 31,92 | 31,58 | 34,16 | 33,77 | 33,19 | 31,5 | 27,97 | 25,74 | 28,92 | 33,23 |
| 20 | 1 | 32,32 | 31,69 | 31,06 | 30,17 | 31,51 | 28,28 | 27,75 | 29,6 | 26,61 | 28,05 | 32,9 | 33,23 |
| 21 | 1 | 32,21 | 28,62 | 31,18 | 31,85 | 30,53 | 32,89 | 32,66 | 30,96 | 29,79 | 31,86 | 29,08 | 29,83 |
| 22 | 1 | 32,85 | 32,46 | 31,1 | 30,17 | 30,48 | 32,55 | 31,14 | 30,78 | 30,23 | 31,2 | 31,59 | 35,14 |
| 23 | 0 | 31,98 | 30,55 | 30,32 | 29,5 | 32,93 | 33,36 | 34,64 | 31,52 | 30,6 | 27,17 | 30,29 | 31,42 |
| 24 | 1 | 31,23 | 31,29 | 30,42 | 30,52 | 30,43 | 32,95 | 31,83 | 31,47 | 30,14 | 27,97 | 30,38 | 33,23 |
| 25 | 1 | 29,01 | 28,92 | 29,71 | 29,91 | 31,14 | 28,03 | 30,26 | 30,93 | 28,8 | 30,7 | 29,46 | 31,61 |
| 26 | 0 | 34,3 | 30,33 | 32,29 | 31,81 | 32,06 | 33,03 | 32,92 | 33,45 | 32,88 | 30,99 | 31,61 | 33,59 |
| 27 | 0 | 34,19 | 32,38 | 29,88 | 33,53 | 32,83 | 35,88 | 33,75 | 32,4 | 30,22 | 28,07 | 33,01 | 34,9 |
| 28 | 1 | 29,54 | 26,44 | 27,33 | 25,72 | 28,36 | 28,2 | 28,46 | 30,84 | 29,36 | 24,08 | 29,15 | 31,81 |
| 29 | 0 | 34,1 | 31,9 | 30,63 | 30,64 | 29,33 | 32,61 | 32,14 | 29,57 | 31,63 | 28,29 | 30,51 | 31,36 |
| 30 | 0 | 32,61 | 29,06 | 31,72 | 32,04 | 30,29 | 31,95 | 33,95 | 29,9 | 30,15 | 28,87 | 32,18 | 30,24 |

0= OD, right eye; 1= OS, left eye; s1-s12, sector 1-12.

S1G Table. 3 PM sectorial vessel density for superficial vascular plexus

| **Patient** | **OD/**  **OS** | **s1** | **s2** | **s3** | **s4** | **s5** | **s6** | **s7** | **s8** | **s9** | **s10** | **s11** | **s12** |
| --- | --- | --- | --- | --- | --- | --- | --- | --- | --- | --- | --- | --- | --- |
| 1 | 1 | 35,78 | 34,51 | 32,88 | 32,1 | 33,33 | 35,29 | 32,15 | 32,64 | 32,25 | 31,67 | 32,65 | 37,28 |
| 2 | 0 | 26,87 | 29,37 | 28,12 | 26,4 | 28,4 | 31,54 | 32 | 32,73 | 29,63 | 28,25 | 29,58 | 27,1 |
| 3 | 1 | 28,69 | 30,08 | 32,15 | 30,58 | 31,14 | 30,84 | 31,3 | 31,85 | 30,23 | 29,43 | 28,72 | 30,2 |
| 4 | 1 | 31,87 | 34,93 | 32,95 | 33,03 | 34,16 | 36,02 | 35,34 | 34,12 | 33,12 | 31,48 | 33,71 | 34,58 |
| 5 | 1 | 30,93 | 28,31 | 26,64 | 29,19 | 31,42 | 29,58 | 32,71 | 31,86 | 31,44 | 27,11 | 30,46 | 30,73 |
| 6 | 0 | 32,38 | 33,03 | 33,81 | 34,79 | 29,5 | 31,77 | 32,41 | 33,42 | 30,94 | 31,14 | 31,67 | 32,84 |
| 7 | 0 | 32,79 | 31,41 | 31,51 | 30,56 | 31,26 | 32,38 | 33,38 | 31,49 | 30,68 | 28,1 | 31,06 | 33,69 |
| 8 | 0 | 29,73 | 28,19 | 26,23 | 27,3 | 27,27 | 31,03 | 30,17 | 30,1 | 25,98 | 23,96 | 28,03 | 29,99 |
| 9 | 1 | 28,73 | 28,13 | 26,64 | 30,72 | 32,89 | 30,52 | 34,46 | 30,13 | 28,58 | 30,03 | 29,79 | 30,25 |
| 10 | 0 | 29,44 | 27,58 | 27,55 | 25,28 | 28,93 | 29,91 | 32,58 | 28,65 | 27,54 | 23,26 | 25,67 | 27,71 |
| 11 | 1 | 34,21 | 31,64 | 34,79 | 32,29 | 32,4 | 32,15 | 29,33 | 30,77 | 28,66 | 28,98 | 30,05 | 33,95 |
| 12 | 0 | 32,61 | 30,68 | 33,19 | 31,14 | 30,5 | 30,43 | 32,78 | 34,57 | 31,45 | 31,84 | 31,51 | 33,36 |
| 13 | 0 | 32,25 | 31,11 | 30,49 | 31,28 | 32,34 | 30,73 | 31,32 | 27,58 | 26,43 | 29,49 | 29,4 | 32,11 |
| 14 | 1 | 30,19 | 30,37 | 28,9 | 29,54 | 28,9 | 31,16 | 31,95 | 30,62 | 27,57 | 27,93 | 30,57 | 30,18 |
| 15 | 1 | 30,45 | 26,27 | 26,83 | 28,18 | 28,17 | 27,79 | 30,04 | 29,16 | 28,82 | 25,3 | 27,21 | 27,45 |
| 16 | 1 | 31,82 | 31,58 | 29,23 | 30,9 | 32,47 | 33,25 | 30,87 | 31,27 | 28,6 | 28,09 | 32,25 | 32,59 |
| 17 | 0 | 31,93 | 34,33 | 32,23 | 33,89 | 32,25 | 33,66 | 33,87 | 32,95 | 31,56 | 30,64 | 33,59 | 31,32 |
| 18 | 1 | 33,77 | 32,61 | 32,47 | 30,2 | 33,88 | 31,75 | 33,31 | 32,21 | 30,52 | 29,33 | 32,75 | 33,84 |
| 19 | 1 | 31,47 | 32,55 | 31,29 | 30,27 | 33,73 | 33,62 | 30,53 | 27,9 | 25,02 | 27,06 | 29,64 | 32,85 |
| 20 | 1 | 28,86 | 27,86 | 28,47 | 31,1 | 32,84 | 30,74 | 29,2 | 31,82 | 24,53 | 22,71 | 28,33 | 30,26 |
| 21 | 1 | 29,77 | 28,46 | 31,49 | 32,48 | 30,71 | 31,53 | 31,7 | 31,55 | 30,19 | 31,1 | 27,62 | 28,9 |
| 22 | 1 | 32,12 | 31,92 | 29,24 | 29,32 | 32,47 | 32,07 | 32,87 | 32,14 | 30,03 | 30,38 | 30,19 | 33,49 |
| 23 | 0 | 34,23 | 31,54 | 31,52 | 29,92 | 33,35 | 34,71 | 34,12 | 32,3 | 30,37 | 28,36 | 31,85 | 33,51 |
| 24 | 1 | 32,43 | 31,49 | 30,9 | 30,53 | 30,75 | 33,19 | 32,54 | 30,68 | 30,82 | 28,65 | 30,67 | 32,45 |
| 25 | 1 | 29,56 | 30,86 | 30,01 | 32,77 | 32,78 | 29,86 | 30,5 | 30,83 | 31,93 | 30,38 | 30,72 | 31,77 |
| 26 | 0 | 33,55 | 30,18 | 31,66 | 31,38 | 31,7 | 32,99 | 32,47 | 31,95 | 31,92 | 31,02 | 31,41 | 32,56 |
| 27 | 0 | 35,48 | 34,65 | 32,07 | 33,85 | 33,81 | 34,64 | 35,55 | 33,25 | 31,98 | 30,8 | 33,11 | 35,81 |
| 28 | 1 | 30,58 | 27,16 | 26,19 | 26,49 | 28,67 | 28,6 | 28,86 | 29,5 | 28,77 | 24,66 | 28,65 | 30,63 |
| 29 | 0 | 29,61 | 25,43 | 17,69 | 25,9 | 25,09 | 28,46 | 31,44 | 30,23 | 33,53 | 28,95 | 30,53 | 30,35 |
| 30 | 0 | 32,96 | 30,72 | 33,16 | 34,41 | 31,06 | 31,76 | 31,93 | 26,41 | 25,37 | 27,06 | 30,26 | 29,07 |

0= OD, right eye; 1= OS, left eye; s1-s12, sector 1-12.

S1H Table. 9 PM sectorial vessel density for superficial vascular plexus

| **Patient** | **OD/**  **OS** | **s1** | **s2** | **s3** | **s4** | **s5** | **s6** | **s7** | **s8** | **s9** | **s10** | **s11** | **s12** |
| --- | --- | --- | --- | --- | --- | --- | --- | --- | --- | --- | --- | --- | --- |
| 1 | 1 | 34,78 | 32,43 | 32,34 | 31,39 | 31,95 | 33,87 | 31,98 | 32,2 | 31,49 | 30,83 | 31,14 | 36,76 |
| 2 | 0 | 30,57 | 32,73 | 27,68 | 27,59 | 29,51 | 30,94 | 31,77 | 30,85 | 28,66 | 27,16 | 30,81 | 30,35 |
| 3 | 1 | 30,2 | 30,38 | 32,06 | 30,02 | 33 | 30,97 | 31,16 | 31,51 | 29,49 | 28,78 | 27,86 | 29,1 |
| 4 | 1 | 31,69 | 32,33 | 32,38 | 33,27 | 32,29 | 34,02 | 33,92 | 33,03 | 30,82 | 30,1 | 32,05 | 33,14 |
| 5 | 1 | 31,55 | 29,19 | 27,51 | 28,49 | 33,65 | 30,09 | 33,15 | 32,7 | 30,62 | 25,56 | 29,11 | 29,69 |
| 6 | 0 | 32,96 | 33,82 | 33,99 | 35,51 | 26,94 | 33,19 | 33,58 | 34,16 | 30,73 | 28,76 | 31,87 | 33,98 |
| 7 | 0 | 34,18 | 32,52 | 32,14 | 30,91 | 32,21 | 32,91 | 34,21 | 32,25 | 30,85 | 28,53 | 31,52 | 33,98 |
| 8 | 0 | 28,5 | 25,17 | 24,78 | 25,37 | 28,14 | 30,79 | 28,55 | 28,6 | 28,4 | 27,34 | 28,97 | 30,59 |
| 9 | 1 | 28,94 | 28,96 | 25,06 | 28,54 | 30,84 | 29,2 | 32,59 | 28,81 | 27,85 | 27,94 | 29,55 | 30,51 |
| 10 | 0 | 28,71 | 27,39 | 27,04 | 26,58 | 30,64 | 30,12 | 33,97 | 30,68 | 27,84 | 23,85 | 26,56 | 29,08 |
| 11 | 1 | 29,14 | 25,08 | 30,6 | 32,24 | 32,53 | 34,17 | 32,01 | 30,18 | 29,49 | 29,31 | 29,72 | 29,1 |
| 12 | 0 | 33,28 | 32,23 | 30,43 | 30,93 | 31,82 | 32,13 | 33,92 | 34,71 | 32,68 | 31,56 | 31,38 | 33,91 |
| 13 | 0 | 33,67 | 32,62 | 30,33 | 31,07 | 31,45 | 31,88 | 33,33 | 31,09 | 28,68 | 30,32 | 29,82 | 31,25 |
| 14 | 1 | 32,04 | 32,62 | 29,33 | 31,68 | 29,85 | 32,33 | 30,86 | 30,19 | 28,95 | 29,83 | 31,3 | 32,52 |
| 15 | 1 | 31,43 | 28,94 | 28,09 | 26,25 | 29,33 | 29,92 | 28,11 | 29,55 | 28,01 | 26,04 | 26,64 | 28,42 |
| 16 | 1 | 30,45 | 30,42 | 29,3 | 32,68 | 32,9 | 33,73 | 31,92 | 31,82 | 28,53 | 29,58 | 31,84 | 32,6 |
| 17 | 0 | 32,94 | 34,27 | 34,71 | 32,94 | 33,05 | 34,01 | 33,11 | 33,2 | 29,58 | 28,77 | 34,29 | 33,22 |
| 18 | 1 | 31,65 | 30,26 | 31,34 | 30,74 | 33,97 | 31,69 | 33,17 | 32,49 | 29,82 | 29,4 | 31,76 | 31,93 |
| 19 | 1 | 32,05 | 33,08 | 31,55 | 30,24 | 34,18 | 35,82 | 33,98 | 32,33 | 28,89 | 29,42 | 33,2 | 34,22 |
| 20 | 1 | 28,31 | 30,55 | 31,11 | 31,54 | 32,44 | 29,51 | 29,76 | 32,21 | 27,12 | 29,46 | 31,1 | 30,49 |
| 21 | 1 | 32,93 | 31,04 | 33,08 | 32,62 | 30,08 | 31,56 | 30,92 | 26,64 | 26,38 | 30,05 | 30,17 | 31,51 |
| 22 | 1 | 30,82 | 32,68 | 29,18 | 31,11 | 31,13 | 29,42 | 28,9 | 30,39 | 30,23 | 30,45 | 30,1 | 33,04 |
| 23 | 0 | 33,44 | 32,96 | 33,11 | 30,33 | 31,22 | 32,92 | 34,19 | 32,47 | 31,76 | 29,53 | 32,07 | 32,74 |
| 24 | 1 | 30,19 | 30,49 | 29,9 | 29,39 | 30,06 | 33,76 | 31,59 | 30,76 | 30,2 | 28,48 | 30,03 | 31,98 |
| 25 | 1 | 30,77 | 31,44 | 31,89 | 31,68 | 34,93 | 30,49 | 31,55 | 31,59 | 31,1 | 30,6 | 30,56 | 34,41 |
| 26 | 0 | 34,55 | 30,79 | 30,71 | 32,6 | 33,16 | 33,29 | 33,61 | 32,15 | 33,88 | 30,76 | 31,97 | 34,39 |
| 27 | 0 | 34,63 | 31,77 | 29,36 | 32,22 | 33,1 | 33,69 | 34,4 | 32,2 | 30,15 | 28,66 | 32,35 | 33,6 |
| 28 | 1 | 26,8 | 28,41 | 29,47 | 28,97 | 29,81 | 31,26 | 30,46 | 31,31 | 29,69 | 26,44 | 30,29 | 28,92 |
| 29 | 0 | 32,71 | 30,28 | 29,37 | 32,41 | 31,64 | 32,49 | 32,15 | 30,94 | 32,95 | 28,66 | 30,7 | 30,55 |
| 30 | 0 | 31,66 | 29,26 | 31,86 | 31,69 | 31,72 | 31,18 | 32,23 | 29,24 | 29,85 | 28,87 | 32,68 | 28,65 |

0= OD, right eye; 1= OS, left eye; s1-s12, sector 1-12.

S1I Table. 9 AM sectorial vessel density for intermediate capillary plexus

| **Patient** | **OD/**  **OS** | **s1** | **s2** | **s3** | **s4** | **s5** | **s6** | **s7** | **s8** | **s9** | **s10** | **s11** | **s12** |
| --- | --- | --- | --- | --- | --- | --- | --- | --- | --- | --- | --- | --- | --- |
| 1 | 1 | 24,41 | 24,43 | 26,74 | 25,3 | 24,16 | 22,26 | 21,73 | 20,67 | 22,18 | 24,65 | 21,83 | 23,46 |
| 2 | 0 | 17,3 | 18,1 | 20,09 | 22,39 | 20,33 | 21,06 | 20,36 | 20,53 | 23,5 | 21,7 | 20,35 | 17,25 |
| 3 | 1 | 18,31 | 20,79 | 22,65 | 21,26 | 21,68 | 20,86 | 21,7 | 21,93 | 23,32 | 22,75 | 18,76 | 15,66 |
| 4 | 1 | 23,07 | 25,43 | 25,73 | 24,3 | 23,28 | 21,89 | 23,8 | 24,83 | 23,6 | 24,52 | 23,37 | 22,93 |
| 5 | 1 | 23,37 | 22,09 | 24,07 | 24,11 | 23,52 | 22,91 | 23,21 | 22,96 | 23,85 | 23,83 | 23,47 | 23,5 |
| 6 | 0 | 23,24 | 25,05 | 24,95 | 25,94 | 24,53 | 22,35 | 22,99 | 22,69 | 24,17 | 23,47 | 24,47 | 23,24 |
| 7 | 0 | 24,16 | 23 | 25,66 | 25,55 | 23,58 | 23,85 | 24,5 | 22,66 | 23,91 | 21,87 | 25,05 | 24,36 |
| 8 | 0 | 22,71 | 21,39 | 22,99 | 24,34 | 22,37 | 20,91 | 21,71 | 21,64 | 22,27 | 24,33 | 23,53 | 21,64 |
| 9 | 1 | 22,63 | 22,03 | 26,67 | 24,89 | 24,12 | 22,81 | 24,81 | 24,79 | 24,49 | 23,28 | 23,57 | 22,89 |
| 10 | 0 | 24,6 | 25,35 | 23,82 | 22,9 | 18,4 | 16,45 | 18,77 | 18,59 | 25,28 | 26,29 | 24,34 | 25,3 |
| 11 | 1 | 24,76 | 24,87 | 27,87 | 28,8 | 19,42 | 19,35 | 18,74 | 19,77 | 26,12 | 26,23 | 25,43 | 25,38 |
| 12 | 0 | 24,22 | 22,59 | 26,18 | 26,09 | 24,34 | 24,27 | 24,36 | 24,26 | 24,83 | 25,26 | 22,43 | 23,3 |
| 13 | 0 | 25,19 | 24,7 | 25,56 | 25,58 | 24,47 | 25,83 | 23,48 | 24,56 | 24,61 | 24,85 | 24,04 | 24,46 |
| 14 | 1 | 16,01 | 17,78 | 16,96 | 18,14 | 18,32 | 20,6 | 22,59 | 20,14 | 22,85 | 22,82 | 22,22 | 19,46 |
| 15 | 1 | 18,95 | 19,91 | 22,07 | 19,9 | 17,62 | 12,67 | 13,09 | 15,52 | 20,31 | 19,52 | 19,41 | 16,22 |
| 16 | 1 | 23,19 | 24,55 | 24,01 | 22,89 | 23,32 | 22,13 | 21,76 | 22,84 | 21,05 | 23,38 | 22,36 | 22,88 |
| 17 | 0 | 22 | 22,04 | 25,43 | 25,29 | 19,39 | 20,57 | 22,08 | 20,67 | 25,41 | 24,89 | 23,1 | 21,32 |
| 18 | 1 | 22,2 | 21,29 | 22,26 | 24,74 | 23,08 | 22,28 | 23,19 | 22,86 | 25,13 | 24,72 | 22,78 | 22,45 |
| 19 | 1 | 21,41 | 23,43 | 25,27 | 24,75 | 23 | 22,08 | 21,25 | 20,98 | 22,13 | 21,61 | 19,91 | 21,66 |
| 20 | 1 | 24,71 | 23,7 | 26,7 | 25,39 | 25,08 | 20,9 | 20,85 | 23,31 | 23,42 | 22,28 | 23,88 | 23,96 |
| 21 | 1 | 21,49 | 20,43 | 19,99 | 22,33 | 22,62 | 24,08 | 22,64 | 24,09 | 23,66 | 23,4 | 20,89 | 21,2 |
| 22 | 1 | 24,53 | 23,77 | 23,04 | 23,82 | 24,64 | 23,83 | 21,35 | 22,39 | 23,74 | 26,17 | 25,42 | 22,72 |
| 23 | 0 | 22,44 | 24,42 | 25,68 | 24,17 | 22,76 | 22,62 | 24,3 | 23,21 | 24,06 | 24,94 | 23,64 | 22,52 |
| 24 | 1 | 23,6 | 23,16 | 23,62 | 24,05 | 23,81 | 24,53 | 23,04 | 23,89 | 26,49 | 26,9 | 25,2 | 24,97 |
| 25 | 1 | 23,69 | 24,16 | 24,42 | 22,34 | 22,69 | 22,89 | 21,73 | 23,82 | 23,35 | 23,04 | 23,86 | 24,4 |
| 26 | 0 | 23,69 | 20,59 | 24,85 | 23,93 | 24,09 | 23,41 | 24,05 | 23,48 | 23,12 | 24,81 | 22,93 | 23,83 |
| 27 | 0 | 25,45 | 27,15 | 24,77 | 26,11 | 24,95 | 26,52 | 24,98 | 25,15 | 24,55 | 25,22 | 25,02 | 25,87 |
| 28 | 1 | 19,09 | 18,74 | 21,58 | 21,45 | 21,35 | 20,47 | 19,88 | 21,76 | 21,8 | 21,22 | 20,44 | 20,63 |
| 29 | 0 | 25,39 | 24,68 | 23,73 | 19,82 | 18,62 | 20,9 | 24,71 | 24,66 | 25,42 | 24,71 | 24,74 | 24,21 |
| 30 | 0 | 23,47 | 25,58 | 24,6 | 25,66 | 22,43 | 22,89 | 24,57 | 23,87 | 24,2 | 23,72 | 24,27 | 23,9 |

0= OD, right eye; 1= OS, left eye; s1-s12, sector 1-12.

S1J Table. 3 PM sectorial vessel density for intermediate capillary plexus

| **Patient** | **OD/**  **OS** | **s1** | **s2** | **s3** | **s4** | **s5** | **s6** | **s7** | **s8** | **s9** | **s10** | **s11** | **s12** |
| --- | --- | --- | --- | --- | --- | --- | --- | --- | --- | --- | --- | --- | --- |
| 1 | 1 | 26,14 | 26,4 | 25,96 | 25,46 | 24,58 | 23,99 | 22,4 | 23,65 | 24,05 | 24,61 | 24,41 | 25,94 |
| 2 | 0 | 15,49 | 18,33 | 20,87 | 20,66 | 20,1 | 21,17 | 20,7 | 20,27 | 20,16 | 21,8 | 20,1 | 15,72 |
| 3 | 1 | 17,59 | 19,36 | 25,32 | 23,03 | 20,65 | 20,66 | 20,54 | 20,57 | 23,38 | 23,78 | 22,37 | 20,81 |
| 4 | 1 | 23,12 | 23,87 | 25,83 | 24,84 | 22,76 | 23,9 | 24,33 | 25,39 | 23,21 | 25,86 | 24,06 | 23,98 |
| 5 | 1 | 22,38 | 21,26 | 24,3 | 23,84 | 20,4 | 21,58 | 22,16 | 23,49 | 22,45 | 21,04 | 22,73 | 23,09 |
| 6 | 0 | 23,29 | 24,12 | 25,14 | 23,52 | 22,98 | 22,57 | 23,26 | 24,89 | 24,83 | 24,08 | 26,06 | 24,36 |
| 7 | 0 | 21,8 | 21,33 | 24,72 | 25,58 | 24,12 | 24,35 | 26,03 | 24,11 | 24,31 | 22,63 | 22,25 | 20,93 |
| 8 | 0 | 24,25 | 21,62 | 20,75 | 25,01 | 22,44 | 22,23 | 22,14 | 23,95 | 24,13 | 24,24 | 23,49 | 23,44 |
| 9 | 1 | 21,45 | 21,85 | 22,62 | 24,75 | 24,11 | 23,43 | 25,74 | 23,69 | 24,71 | 24,02 | 21,86 | 22,15 |
| 10 | 0 | 24,74 | 24,62 | 22,95 | 19,28 | 20,38 | 22,43 | 23,89 | 22,18 | 23,1 | 24,64 | 23,33 | 25,99 |
| 11 | 1 | 24,19 | 23,56 | 26,73 | 23,68 | 23,98 | 22 | 19,9 | 22,83 | 25,74 | 26,25 | 24,92 | 24,92 |
| 12 | 0 | 25 | 25,02 | 25,97 | 25,83 | 25,19 | 25,56 | 25,16 | 25,74 | 24,56 | 25,07 | 22,99 | 24,59 |
| 13 | 0 | 22,27 | 22,71 | 26,01 | 25,35 | 24,85 | 22,43 | 20,91 | 21 | 20,22 | 23,36 | 22,58 | 23,56 |
| 14 | 1 | 20,49 | 18,7 | 21,19 | 18,96 | 19,26 | 21,45 | 22,4 | 21,79 | 23,31 | 22,19 | 21,1 | 20,72 |
| 15 | 1 | 20,61 | 19,07 | 19,07 | 20,94 | 21,5 | 20,82 | 19,87 | 19,96 | 21,38 | 19,64 | 19,93 | 21,31 |
| 16 | 1 | 24,07 | 24,17 | 22,94 | 24,31 | 23,02 | 22,66 | 18,44 | 20,51 | 20,56 | 22,48 | 23,05 | 23,53 |
| 17 | 0 | 18,07 | 21,55 | 25,26 | 27,94 | 23,3 | 23,05 | 23,56 | 21,96 | 24,6 | 23,58 | 22,1 | 18,74 |
| 18 | 1 | 24,19 | 23,05 | 23,36 | 23,97 | 23 | 23,61 | 23,99 | 24,14 | 24,88 | 23,97 | 23,25 | 23,54 |
| 19 | 1 | 24,74 | 23,3 | 22,45 | 25,72 | 24,1 | 22,66 | 17,02 | 16,34 | 19,25 | 22,03 | 20,99 | 22,56 |
| 20 | 1 | 21,11 | 22,4 | 22,68 | 24,91 | 26,1 | 23,68 | 21,7 | 23,54 | 22,39 | 13,77 | 19,21 | 19,69 |
| 21 | 1 | 20,19 | 19,6 | 21,21 | 22,96 | 20,75 | 23,06 | 22,33 | 24,55 | 24,15 | 22,55 | 19,32 | 18,82 |
| 22 | 1 | 23,25 | 23,23 | 24,43 | 23,95 | 22,75 | 23,69 | 23,49 | 24,1 | 24,49 | 25,17 | 24,44 | 22,09 |
| 23 | 0 | 21,24 | 21,66 | 24,95 | 21,98 | 23,51 | 22,01 | 23,99 | 24,53 | 24,08 | 23,64 | 24,02 | 23,75 |
| 24 | 1 | 24,5 | 21,49 | 24,26 | 23,58 | 22,77 | 22,58 | 22,85 | 22,67 | 26,18 | 24,73 | 23,71 | 23,84 |
| 25 | 1 | 22,34 | 22,42 | 24,14 | 22,86 | 21,02 | 21,52 | 21,07 | 23,13 | 23,75 | 22,37 | 21,71 | 21,23 |
| 26 | 0 | 23,52 | 22,04 | 24,4 | 25,11 | 24,84 | 23,45 | 23,98 | 23,32 | 22,62 | 22,75 | 22,53 | 23,89 |
| 27 | 0 | 25,59 | 26,94 | 26,47 | 27,03 | 25,91 | 27,27 | 27,26 | 24,99 | 25,88 | 25,11 | 24,78 | 25,68 |
| 28 | 1 | 19,76 | 20,56 | 21,79 | 19,63 | 19,52 | 19,09 | 19,06 | 21,05 | 21,69 | 20,69 | 20,84 | 19,83 |
| 29 | 0 | 17,2 | 14,21 | 9,96 | 16,32 | 13,63 | 15,32 | 19,3 | 21,76 | 24,99 | 24,64 | 22,07 | 21,4 |
| 30 | 0 | 24,88 | 26,6 | 27,04 | 26,32 | 24,54 | 23,47 | 21,76 | 19,14 | 19,98 | 19,11 | 22,51 | 21,98 |

0= OD, right eye; 1= OS, left eye; s1-s12, sector 1-12.

S1K Table. 9 PM sectorial vessel density for intermediate capillary plexus

| **Patient** | **OD/**  **OS** | **s1** | **s2** | **s3** | **s4** | **s5** | **s6** | **s7** | **s8** | **s9** | **s10** | **s11** | **s12** |
| --- | --- | --- | --- | --- | --- | --- | --- | --- | --- | --- | --- | --- | --- |
| 1 | 1 | 25,93 | 25,68 | 24,28 | 25,17 | 24,63 | 22 | 22,08 | 22,91 | 22,9 | 23,33 | 21,83 | 24,52 |
| 2 | 0 | 21,74 | 22,45 | 21,71 | 19,35 | 20,93 | 21,63 | 20,89 | 21,21 | 19,97 | 20,69 | 20,54 | 19,91 |
| 3 | 1 | 21,73 | 21,49 | 23,77 | 23,4 | 24,26 | 21,35 | 23,32 | 23,33 | 24,91 | 23,04 | 21,67 | 22,23 |
| 4 | 1 | 22,33 | 23,48 | 23,99 | 24,17 | 21,92 | 21,9 | 22,69 | 23,68 | 23,12 | 25,9 | 22,11 | 21,48 |
| 5 | 1 | 23,19 | 22,68 | 24,8 | 25,62 | 21,39 | 22,04 | 22,01 | 23,88 | 24,25 | 23,41 | 22,01 | 21,84 |
| 6 | 0 | 23,37 | 25,44 | 24,3 | 24,71 | 18,39 | 20,91 | 21,71 | 23,42 | 25,61 | 23,2 | 25,31 | 24,26 |
| 7 | 0 | 23,65 | 22,85 | 24,82 | 26,07 | 23,51 | 24,29 | 25,07 | 24,06 | 24,57 | 24,14 | 24,8 | 24,49 |
| 8 | 0 | 22,46 | 19,86 | 22,74 | 23,07 | 22,87 | 21,75 | 20,68 | 21,33 | 24,87 | 23,13 | 24,98 | 23,76 |
| 9 | 1 | 21,14 | 21,72 | 24,65 | 26,52 | 23,31 | 22,8 | 24,43 | 23,44 | 23,86 | 22,69 | 20,74 | 20,91 |
| 10 | 0 | 24,03 | 25,52 | 24,29 | 23,51 | 25,28 | 23,98 | 24,43 | 24,62 | 25,97 | 24,24 | 23,64 | 25,27 |
| 11 | 1 | 20,18 | 13,81 | 23,39 | 25,05 | 24,16 | 26,78 | 24,76 | 26,35 | 26,41 | 27,02 | 22,37 | 19,61 |
| 12 | 0 | 25,82 | 25,66 | 27,53 | 26,66 | 25,67 | 26,59 | 25,5 | 24,91 | 24,01 | 24,09 | 23,39 | 24,42 |
| 13 | 0 | 23,46 | 23,5 | 25,62 | 25,89 | 24,1 | 24,43 | 24,63 | 24,05 | 23,57 | 24,27 | 22,27 | 22,96 |
| 14 | 1 | 22,54 | 22,49 | 23,08 | 22,4 | 21,36 | 22,98 | 23,58 | 23,02 | 25,83 | 25,15 | 24,02 | 23,64 |
| 15 | 1 | 21,77 | 21,65 | 21,39 | 20,13 | 21,91 | 21,58 | 18,83 | 19,09 | 20,8 | 20,65 | 19,39 | 20,38 |
| 16 | 1 | 22,87 | 23,08 | 23,83 | 22,55 | 23,32 | 20,68 | 19,65 | 20,85 | 20,08 | 24,33 | 23,91 | 23,12 |
| 17 | 0 | 18,81 | 21,14 | 26,23 | 25,05 | 20,94 | 22,9 | 21,96 | 22,83 | 22,25 | 21,98 | 23,06 | 20,35 |
| 18 | 1 | 22,17 | 20,9 | 23,2 | 23,22 | 24,22 | 24,87 | 24,98 | 23,7 | 25,21 | 24,95 | 23,48 | 24,13 |
| 19 | 1 | 23,29 | 22,22 | 22,52 | 24,01 | 22,94 | 23,76 | 22,29 | 22,64 | 23,51 | 23,67 | 23,17 | 23,02 |
| 20 | 1 | 21,3 | 22,69 | 26,38 | 25,06 | 26,75 | 24,87 | 24,61 | 25,71 | 23,72 | 22,39 | 22,44 | 21,18 |
| 21 | 1 | 23,7 | 22,54 | 23,7 | 24,19 | 20,56 | 21,16 | 21,4 | 19,71 | 19,88 | 22,31 | 21,76 | 21,64 |
| 22 | 1 | 22,36 | 23,48 | 24,52 | 24,88 | 22,99 | 22,44 | 19,86 | 21,83 | 24,71 | 24,6 | 24,07 | 21,11 |
| 23 | 0 | 21,17 | 23,6 | 26,99 | 23,49 | 20,78 | 18,98 | 22,84 | 22,09 | 23,44 | 24,37 | 22,38 | 21,77 |
| 24 | 1 | 23,94 | 21,76 | 24,74 | 24,6 | 25,09 | 24,3 | 24,24 | 24,21 | 26,71 | 25,29 | 24,89 | 23,87 |
| 25 | 1 | 25,5 | 25,89 | 26,05 | 24,13 | 24,37 | 23,3 | 21,71 | 23,33 | 25,63 | 23,82 | 23,85 | 24,98 |
| 26 | 0 | 23,22 | 22,47 | 23,4 | 25,65 | 23,63 | 24,14 | 24,63 | 23,42 | 23,39 | 23,34 | 23,37 | 24,44 |
| 27 | 0 | 25,92 | 26,23 | 25,27 | 25,29 | 24,56 | 25,34 | 24,25 | 24,01 | 25,45 | 25,69 | 24,97 | 25,04 |
| 28 | 1 | 14,17 | 18,55 | 22,44 | 21,39 | 20,96 | 19,91 | 21,2 | 22,58 | 23,32 | 23,35 | 19,86 | 16,61 |
| 29 | 0 | 23,12 | 19,9 | 18,81 | 23,72 | 22,69 | 23,04 | 24,87 | 23,43 | 25,89 | 24,76 | 25,36 | 23,39 |
| 30 | 0 | 22,53 | 23,88 | 26,44 | 24,04 | 25,02 | 23,92 | 23,91 | 23,27 | 24,44 | 23,5 | 22,2 | 21,65 |

0= OD, right eye; 1= OS, left eye; s1-s12, sector 1-12.

S1L Table. 9 AM sectorial vessel density for deep capillary plexus

| **Patient** | **OD/**  **OS** | **s1** | **s2** | **s3** | **s4** | **s5** | **s6** | **s7** | **s8** | **s9** | **s10** | **s11** | **s12** |
| --- | --- | --- | --- | --- | --- | --- | --- | --- | --- | --- | --- | --- | --- |
| 1 | 1 | 25,73 | 25,56 | 28,33 | 26,62 | 27,32 | 26,07 | 22,67 | 22,14 | 24,98 | 26,71 | 21,79 | 24,79 |
| 2 | 0 | 17,29 | 19,36 | 22,27 | 22,39 | 21,61 | 23,08 | 21,79 | 21,62 | 22,78 | 22,36 | 22,09 | 19,93 |
| 3 | 1 | 26,02 | 26,98 | 29,28 | 26,88 | 26,53 | 24,11 | 25,19 | 23,41 | 26,79 | 25,82 | 26,06 | 24,26 |
| 4 | 1 | 22,96 | 25,31 | 25,75 | 28,16 | 25,26 | 23,09 | 23,08 | 25,12 | 23,64 | 25,98 | 22,68 | 22,99 |
| 5 | 1 | 27,14 | 27,62 | 24,63 | 23,88 | 23,93 | 25,34 | 23,9 | 23,82 | 23,97 | 23,13 | 26,17 | 27,74 |
| 6 | 0 | 24,33 | 26,98 | 24,69 | 27,34 | 25,71 | 27,61 | 25,23 | 24,43 | 26,86 | 27,86 | 27,63 | 24,49 |
| 7 | 0 | 26,52 | 26,71 | 29,5 | 28,71 | 26,61 | 28,88 | 27,31 | 23,88 | 28,12 | 25,42 | 27,5 | 27,76 |
| 8 | 0 | 23,86 | 21,57 | 23,63 | 27,56 | 26,72 | 28,83 | 27,43 | 27,91 | 23,63 | 25,26 | 24,1 | 22,73 |
| 9 | 1 | 24,64 | 23,7 | 23,61 | 25,07 | 23,92 | 23,35 | 27,97 | 27,67 | 28,21 | 25,77 | 24,27 | 25,48 |
| 10 | 0 | 26,19 | 26,99 | 26,2 | 24,78 | 20,73 | 19,23 | 20,97 | 19,84 | 26,03 | 23,9 | 24,34 | 27,89 |
| 11 | 1 | 24,08 | 24,09 | 28,38 | 29,08 | 23,09 | 24,33 | 21,47 | 19,17 | 26,62 | 26,25 | 23,9 | 24,88 |
| 12 | 0 | 27,15 | 25,5 | 28,08 | 28,28 | 24,24 | 24,55 | 26,27 | 24,92 | 29,34 | 25,97 | 27,23 | 28,97 |
| 13 | 0 | 30,39 | 27,72 | 27,81 | 27,07 | 24,54 | 27,2 | 28,51 | 26,28 | 26,38 | 25,76 | 27,88 | 28,63 |
| 14 | 1 | 14,34 | 13,26 | 13,02 | 14,21 | 16,36 | 18,63 | 19,07 | 19,07 | 19,48 | 21,16 | 21,71 | 17,71 |
| 15 | 1 | 19,32 | 21,71 | 24,73 | 25,42 | 22,19 | 14,6 | 18,03 | 17,35 | 21,88 | 20,37 | 17,41 | 16,54 |
| 16 | 1 | 26,53 | 25,83 | 22,61 | 25,63 | 26,65 | 26,12 | 25,3 | 23,15 | 21,29 | 22,1 | 24,17 | 25,96 |
| 17 | 0 | 22,89 | 23,24 | 24,16 | 24,32 | 20,7 | 22,43 | 21,34 | 21,46 | 26,19 | 23,89 | 23,82 | 23,24 |
| 18 | 1 | 27,58 | 24,8 | 24,57 | 23,69 | 19,45 | 20,21 | 19,87 | 19,18 | 19,88 | 21,61 | 23,96 | 26,47 |
| 19 | 1 | 29,06 | 28,87 | 28,45 | 27,98 | 28,27 | 25,87 | 26,17 | 26,6 | 26,91 | 24,81 | 24,9 | 27,08 |
| 20 | 1 | 28,02 | 24,93 | 27,7 | 28,7 | 26,4 | 23,84 | 23,12 | 26,32 | 26,61 | 27,15 | 29,26 | 28,02 |
| 21 | 1 | 24,63 | 21,34 | 18,31 | 23,92 | 22,29 | 21,49 | 22,31 | 22,44 | 24,23 | 24,48 | 24,94 | 27,63 |
| 22 | 1 | 24,09 | 26,22 | 26,5 | 21,58 | 23,14 | 22,8 | 19,97 | 18,84 | 16,67 | 20,79 | 23,7 | 23,56 |
| 23 | 0 | 20,96 | 24,18 | 25,33 | 24,26 | 24,1 | 24 | 24,34 | 25,86 | 24,69 | 22,64 | 25,31 | 23,04 |
| 24 | 1 | 25,15 | 25,91 | 23,39 | 25,17 | 22,12 | 23,11 | 22,65 | 23,93 | 26,19 | 26,35 | 25,89 | 24,78 |
| 25 | 1 | 26,88 | 25,46 | 23,01 | 25,25 | 24,65 | 24,94 | 25,28 | 24,18 | 23,65 | 24 | 24,94 | 24,89 |
| 26 | 0 | 27,12 | 26,18 | 26,6 | 26,74 | 26,13 | 25,79 | 27,78 | 25,27 | 26,71 | 27,08 | 27,01 | 28,12 |
| 27 | 0 | 23,45 | 24,63 | 21,02 | 23,82 | 23,8 | 26,02 | 25,18 | 22,85 | 22,88 | 24,86 | 24,45 | 23,92 |
| 28 | 1 | 23,98 | 23,66 | 25,65 | 23,22 | 21,46 | 20,26 | 22,65 | 20,4 | 20,4 | 18,61 | 20,14 | 22,41 |
| 29 | 0 | 26,07 | 24,39 | 23,85 | 21,18 | 24,76 | 29,06 | 30,25 | 27,26 | 27,24 | 26,01 | 25,48 | 25,47 |
| 30 | 0 | 24,82 | 25,7 | 26,85 | 27,13 | 22,83 | 23,66 | 22,05 | 22,61 | 24,2 | 23,71 | 24,34 | 24,29 |

0= OD, right eye; 1= OS, left eye; s1-s12, sector 1-12.

S1M Table. 3 PM sectorial vessel density for deep capillary plexus

| **Patient** | **OD/**  **OS** | **s1** | **s2** | **s3** | **s4** | **s5** | **s6** | **s7** | **s8** | **s9** | **s10** | **s11** | **s12** |
| --- | --- | --- | --- | --- | --- | --- | --- | --- | --- | --- | --- | --- | --- |
| 1 | 1 | 31,22 | 29,37 | 29,45 | 28,15 | 25,48 | 24,67 | 22,55 | 23,09 | 25,33 | 26,62 | 26,41 | 32,66 |
| 2 | 0 | 14,84 | 18,64 | 21,77 | 21,56 | 20,62 | 22,33 | 21,61 | 21,4 | 21,23 | 21,3 | 20,65 | 16,98 |
| 3 | 1 | 23,63 | 23,71 | 29,73 | 26,9 | 26,79 | 24,84 | 24,6 | 23,73 | 26,23 | 27,67 | 27,11 | 27,15 |
| 4 | 1 | 23,9 | 24,34 | 25,24 | 26,56 | 25,22 | 26,43 | 25,71 | 26,29 | 25,45 | 25,41 | 24,42 | 24,03 |
| 5 | 1 | 25,6 | 23,91 | 22,61 | 20,97 | 21,49 | 24,52 | 23,96 | 25,92 | 24,34 | 22,94 | 25,64 | 25,83 |
| 6 | 0 | 25,96 | 28,76 | 26,92 | 27,71 | 24,11 | 26,81 | 24,3 | 26,31 | 27,47 | 28,83 | 29,43 | 27,63 |
| 7 | 0 | 23,07 | 23,51 | 27,56 | 27,21 | 26,43 | 30,99 | 29,2 | 26,76 | 28,94 | 26,23 | 24,24 | 22,64 |
| 8 | 0 | 27,17 | 24,89 | 23,13 | 25,98 | 23,9 | 25,07 | 27,08 | 27,3 | 25,73 | 24,94 | 25,32 | 24,97 |
| 9 | 1 | 21,76 | 21,32 | 20,26 | 25,69 | 28,94 | 29,03 | 30,52 | 29,41 | 27 | 23,95 | 19,49 | 19,36 |
| 10 | 0 | 25,94 | 25,3 | 24,95 | 21,78 | 22,89 | 26,9 | 27,5 | 24,86 | 25,14 | 24,29 | 23,78 | 27,1 |
| 11 | 1 | 23,85 | 22,78 | 26,65 | 26,87 | 28,08 | 23,48 | 20,14 | 22,17 | 25,23 | 24,55 | 24,13 | 25,28 |
| 12 | 0 | 27,64 | 26,39 | 28,5 | 28,54 | 26,18 | 28 | 27,55 | 26,68 | 28,36 | 24,32 | 23,54 | 26,75 |
| 13 | 0 | 25,33 | 24,08 | 27,7 | 28,44 | 27,67 | 27,08 | 26,53 | 24,38 | 24,3 | 24,27 | 25,72 | 25,22 |
| 14 | 1 | 17,49 | 15 | 16,59 | 17,53 | 18,7 | 18,93 | 19,21 | 19,6 | 19,83 | 17,25 | 19 | 19,43 |
| 15 | 1 | 21,96 | 23,23 | 23,37 | 24,45 | 24,93 | 23,1 | 23,74 | 22,94 | 22,4 | 21,23 | 19,85 | 23,28 |
| 16 | 1 | 26,46 | 25,43 | 22,43 | 26,18 | 26,06 | 24,88 | 22,95 | 22,01 | 22,47 | 22,15 | 25,07 | 26,84 |
| 17 | 0 | 19,08 | 21,64 | 25,03 | 28,33 | 26,71 | 28,57 | 24,86 | 24,41 | 24,7 | 22,2 | 19,34 | 17,88 |
| 18 | 1 | 25,07 | 23,64 | 23,29 | 22,12 | 21,19 | 23,36 | 23,03 | 20,97 | 19,76 | 20,8 | 22,69 | 25,06 |
| 19 | 1 | 31,58 | 29,98 | 26,66 | 29,24 | 29,1 | 26,36 | 20,17 | 22,93 | 22,96 | 24,15 | 26,35 | 27,34 |
| 20 | 1 | 28,5 | 24,07 | 24,99 | 28,79 | 28,3 | 24,96 | 23,85 | 26,26 | 25,58 | 20,54 | 26,68 | 27,46 |
| 21 | 1 | 25,51 | 21,97 | 20,53 | 23,05 | 21,51 | 19,77 | 22,5 | 23,86 | 24,45 | 25,04 | 24,82 | 25,75 |
| 22 | 1 | 21,88 | 25,73 | 24,08 | 22,01 | 22,34 | 22,36 | 21,63 | 21,73 | 18,82 | 17,71 | 22,35 | 20,82 |
| 23 | 0 | 23,63 | 25,05 | 26,14 | 22,15 | 23,98 | 24,7 | 23,82 | 26,96 | 25,98 | 24,43 | 26,1 | 25,73 |
| 24 | 1 | 28,43 | 28,64 | 27,33 | 25,42 | 20,02 | 20,83 | 19,59 | 20,43 | 24,27 | 24,13 | 24,79 | 26,96 |
| 25 | 1 | 22,73 | 22,96 | 23,26 | 23,59 | 21,82 | 21,89 | 23,12 | 22,44 | 24,08 | 22,95 | 22,46 | 22,3 |
| 26 | 0 | 26,33 | 25,65 | 25,93 | 27,39 | 25,35 | 27,18 | 27,27 | 24,63 | 25,49 | 25,19 | 26,37 | 26,28 |
| 27 | 0 | 25,01 | 26,13 | 23,72 | 25,9 | 25,07 | 26,36 | 27,75 | 23,68 | 24,03 | 25,96 | 25,34 | 24,38 |
| 28 | 1 | 21,58 | 21,74 | 23,74 | 22,49 | 20,83 | 21,57 | 20,95 | 21,05 | 20,93 | 16,08 | 18,75 | 18,46 |
| 29 | 0 | 18,57 | 13,08 | 8,58 | 17,94 | 20,21 | 20,47 | 24,79 | 23,16 | 26,19 | 25,27 | 21,16 | 20,62 |
| 30 | 0 | 26,99 | 27,28 | 27,4 | 26,53 | 22,98 | 24,66 | 20,16 | 20,38 | 20,15 | 21,89 | 23,89 | 25,19 |

0= OD, right eye; 1= OS, left eye; s1-s12, sector 1-12.

S1N Table. 9 PM sectorial vessel density for deep capillary plexus

| **Patient** | **OD/**  **OS** | **s1** | **s2** | **s3** | **s4** | **s5** | **s6** | **s7** | **s8** | **s9** | **s10** | **s11** | **s12** |
| --- | --- | --- | --- | --- | --- | --- | --- | --- | --- | --- | --- | --- | --- |
| 1 | 1 | 31,52 | 28,2 | 26,17 | 26,16 | 25,09 | 21,98 | 23,14 | 23,04 | 23,23 | 27,99 | 26,49 | 30,94 |
| 2 | 0 | 20,42 | 22,28 | 22,46 | 20,14 | 21,02 | 23,84 | 24,67 | 22,8 | 21,54 | 21,87 | 20,56 | 20,96 |
| 3 | 1 | 29,53 | 27,53 | 28,41 | 28,06 | 27,05 | 26,22 | 27,46 | 27,29 | 28,41 | 26,75 | 27,75 | 28,6 |
| 4 | 1 | 25,26 | 26,38 | 23,63 | 26,33 | 21,71 | 20,57 | 21,27 | 23,22 | 22,61 | 25,59 | 22,74 | 25,13 |
| 5 | 1 | 26,59 | 26,75 | 26,4 | 23,94 | 22,38 | 23,29 | 22,11 | 25,95 | 23,53 | 21,87 | 24,62 | 25,52 |
| 6 | 0 | 23,27 | 26,89 | 25,2 | 25,73 | 20,03 | 24,83 | 24,63 | 26,32 | 28,3 | 27,08 | 27,1 | 24,64 |
| 7 | 0 | 26,52 | 25,62 | 28,95 | 27,96 | 27,09 | 30,01 | 28,91 | 26,02 | 28,86 | 27,39 | 29,5 | 27,51 |
| 8 | 0 | 26,12 | 23,85 | 25,33 | 24,97 | 26,52 | 27,63 | 26,53 | 25,72 | 27,09 | 27,44 | 27,43 | 27,19 |
| 9 | 1 | 24,79 | 21,74 | 23,89 | 25,09 | 23,27 | 23,35 | 26,3 | 23,96 | 24,29 | 24,55 | 24,48 | 24,88 |
| 10 | 0 | 26,42 | 26,79 | 27 | 26,21 | 27,7 | 29,46 | 30,4 | 29,2 | 27,66 | 23,88 | 24,98 | 27,86 |
| 11 | 1 | 19,18 | 12,32 | 24,06 | 27,07 | 28,46 | 27,55 | 27,53 | 26,15 | 25,97 | 25,77 | 19,18 | 18,57 |
| 12 | 0 | 26,65 | 26,51 | 29,06 | 29,69 | 25,38 | 26,64 | 27,38 | 26,22 | 27,21 | 24,17 | 23,03 | 27,03 |
| 13 | 0 | 29,7 | 27,43 | 29,89 | 28,07 | 24,73 | 25,26 | 27,42 | 26,03 | 25,3 | 24,09 | 25,93 | 26,87 |
| 14 | 1 | 24,63 | 20,43 | 18,9 | 19,86 | 19,94 | 20,54 | 20,82 | 20,05 | 21,06 | 23,11 | 25,12 | 25,54 |
| 15 | 1 | 23,58 | 23,8 | 23,26 | 22,6 | 24,62 | 23,11 | 22,47 | 21,21 | 22,44 | 20,63 | 19,09 | 21,96 |
| 16 | 1 | 25,11 | 26,1 | 24,51 | 25,65 | 27,57 | 24,99 | 21,99 | 20,69 | 18,34 | 22,58 | 24,35 | 25,66 |
| 17 | 0 | 20,53 | 22,91 | 24,08 | 23,6 | 23,68 | 25,89 | 22,98 | 23,33 | 21,68 | 19,87 | 23,06 | 21,83 |
| 18 | 1 | 25,22 | 21,3 | 21,94 | 22,96 | 22,37 | 24,31 | 23,15 | 22,51 | 22,05 | 21,45 | 24,59 | 24,37 |
| 19 | 1 | 28,76 | 27,67 | 25,7 | 29,28 | 30,66 | 29,3 | 27,1 | 27,98 | 26,59 | 27,33 | 28,78 | 26,82 |
| 20 | 1 | 30,75 | 28,65 | 30,04 | 29,06 | 28,36 | 25,31 | 25,07 | 27,31 | 27 | 25,77 | 26,99 | 27,5 |
| 21 | 1 | 26,14 | 22,14 | 22,79 | 24,82 | 21,08 | 21,42 | 23,46 | 21,07 | 21,57 | 22,85 | 27,42 | 28,47 |
| 22 | 1 | 20,71 | 25,25 | 24,83 | 24,66 | 24,29 | 21,16 | 20,25 | 19,4 | 15,93 | 17,07 | 21,79 | 20,53 |
| 23 | 0 | 20,21 | 22,74 | 26,29 | 22,18 | 19,52 | 20,76 | 22,71 | 26,07 | 24,96 | 24,79 | 25,71 | 22,11 |
| 24 | 1 | 24,48 | 23,25 | 24,11 | 26,15 | 24,18 | 25,51 | 23,98 | 25,44 | 26,48 | 25,85 | 26,33 | 23,53 |
| 25 | 1 | 26,63 | 27,33 | 23,42 | 25,53 | 26,55 | 23,89 | 25,12 | 24,63 | 23,97 | 23,5 | 25,04 | 25,6 |
| 26 | 0 | 27,95 | 27,54 | 25,73 | 27,37 | 26,27 | 27,82 | 27,99 | 25 | 25,99 | 25,95 | 28,3 | 28,79 |
| 27 | 0 | 23,21 | 25,03 | 22,66 | 24,93 | 26,01 | 27,56 | 26,02 | 25,04 | 24,66 | 25,4 | 24,21 | 23,05 |
| 28 | 1 | 19,7 | 22,96 | 26,31 | 23,05 | 20,79 | 19,79 | 20,08 | 20,22 | 21,76 | 19,64 | 22,47 | 19,94 |
| 29 | 0 | 24,03 | 22,07 | 22,71 | 26,52 | 28,99 | 28,32 | 27,72 | 26,2 | 26,36 | 26,4 | 24,77 | 24,21 |
| 30 | 0 | 25,76 | 27,66 | 28,19 | 26,36 | 22,38 | 22,33 | 21,51 | 22,9 | 23,79 | 23,5 | 23,34 | 24,4 |

0= OD, right eye; 1= OS, left eye; s1-s12, sector 1-12.
